# Thromboprophylaxis in the End-of-Life Cancer Care: The Update

**DOI:** 10.3390/cancers12030600

**Published:** 2020-03-05

**Authors:** Ewa Zabrocka, Ewa Sierko

**Affiliations:** 1 Department of Radiation Oncology, Stony Brook University, Stony Brook, New York, NY 11794, USA; ewa.zabrocka1@gmail.com; 2 Department of Oncology, Medical University of Bialystok, 12 Ogrodowa St., 15-027 Bialystok, Poland; 3 Comprehensive Cancer Center in Bialystok, 15-027 Bialystok, Poland

**Keywords:** thromboprophylaxis, venous thromboembolism, cancer, hospice, palliative care units, low molecular weight heparin, deep vein thrombosis, pulmonary embolism

## Abstract

Cancer patients are at increased risk for venous thromboembolism (VTE), which further increases with advanced stages of malignancy, prolonged immobilization, or prior history of thrombosis. To reduce VTE-related mortality, many official guidelines encourage the use of thromboprophylaxis (TPX) in cancer patients in certain situations, e.g., during chemotherapy or in the perioperative period. TPX in the end-of-life care, however, remains controversial. Most recommendations on VTE prophylaxis in cancer patients are based on the outcomes of clinical trials that excluded patients under palliative or hospice care. This translates to the paucity of official guidelines on TPX dedicated to this group of patients. The problem should not be underestimated as VTE is known to be associated with symptoms adversely impacting the quality of life (QoL), i.e., limb or chest pain, dyspnea, hemoptysis. In end-of-life care, where the assurance of the best possible QoL should be the highest priority, VTE prophylaxis may eliminate the symptom burden related to thrombosis. However, large randomized studies determining the benefits and risks profiles of TPX in patients nearing the end of life are lacking. This review summarized available data on TPX in this population, analyzed potential tools for VTE risk prediction in the view of this group of patients, and summarized the most current recommendations on TPX pertaining to terminal care.

## 1. Introduction

Cancer is among well-recognized risk factors for venous thromboembolism (VTE) [1]. The relative risk of VTE in cancer patients compared to patients without cancer ranges between 4 and 7 [2]. The main forms of the thromboembolic disease include pulmonary embolism (PE) and deep vein thrombosis (DVT). Advanced cancer patients are at particularly increased risk for VTE, taking into account their diagnosis and usually poor performance status, resulting in a decreased level of activity or even immobilization [3]. The exact VTE incidence and prevalence in the population of cancer patients under hospice or palliative care have not been well investigated, and available reports are scant. Palliative care physicians have been found to underestimate the prevalence of VTE in hospice inpatients, and, in one study, they estimated the prevalence to be only 1–5% [4]. This is, however, a physician recall estimate, suggesting that VTE in hospice is not perceived as a common clinical problem. In a retrospective study, approximately 10% of 712 patients hospitalized in palliative care units (PCU) were found to have DVT on Doppler echography or PE on either computed tomography or ventilation/perfusion scintigraphy [5], although this likely did not reflect true prevalence since only the patients with clinical suspicion for VTE were tested. In fact, VTE prevalence in this population is most likely higher. Using a diagnostic bilateral femoral vein ultrasonography, White et al. [6] found that DVT involving femoral vein was present in about a third of advanced cancer patients admitted to PCU. In a prospective study by Johnson et al., DVT was found in as many as 52% of hospice inpatients [7]. However, the actual prevalence may be lower since light reflection rheography used to detect DVT in this study cannot distinguish between the external compression of the vein or obstruction of flow by thrombosis. 

In cancer VTE studies, the primary outcome is often survival, whereas, in the palliative and hospice care population, the quality of life (QoL) is the most relevant outcome in clinical practice. The symptom burden associated with VTE, including dyspnea, chest or limb pain, and limb swelling, can adversely affect the QoL, but the reports on the actual symptom profile and severity in hospice and palliative care patient population are conflicting. Although half of the hospice patients in the study by Johnson et al. had radiographic suspicion of DVT, only 9% had VTE symptoms at the time of diagnosis [7]. On the other hand, the study of Soto-Cardenas et al. [5] revealed that half of PCU patients with DVT were suffering from localized pain, and 80% of those with PE reported dyspnea. The results of a recent observational study by White et al. [6] do not support these findings. Among the signs and symptoms of VTE, including limb pain, chest pain, breathlessness, hemoptysis, and lower extremity edema, only the latter is significantly more often found in PCU patients with DVT, indicating that symptom burden attributed to VTE may be, in fact, overestimated in this patient population. Aside from the physical aspect, VTE can also be a source of significant psychological distress, which by some cancer patients has been described as even worse than their cancer experiences [8]. Symptom control remains the mainstay of palliative care; therefore, symptom burden caused by VTE warrants the discussion on primary and secondary thromboprophylaxis in patients approaching the end of life.

Thromboprophylaxis is recommended for hospitalized cancer patients who do not have contraindications to such therapy [9]. However, it has not been commonly used in palliative and hospice care patients, which may result from the lack of official recommendations in that matter. Clinical trials investigating VTE prophylaxis in the population of cancer patients usually exclude palliative or hospice care patients [10]. Ethical factors likely play a major role in this approach since thromboprophylaxis (TPX) may be perceived as one of the ways of postponing the natural death, which would not be in line with the philosophy and foundations of palliative care. Nevertheless, it still remains questionable whether TPX can affect life expectancy in this group of patients. One prospective randomized study showed no statistically significant survival benefit of prophylactic nadroparin in hospitalized palliative care patients with an estimated life expectancy of ≤6 months [11]; however, only 20 patients were enrolled in the study. Another challenging aspect is also the degree of symptom relief by TPX, which is difficult to estimate due to the lack of standardized tools for QoL assessment. All these uncertainties seem to have a substantial impact on health care providers’ decisions on prescribing anticoagulation for palliative care and hospice patients. The usefulness of TPX is also challenged by a recent observational study by White et al. [6], who investigated the prevalence and symptom burden of DVT as well as its association with TPX in 273 PCU patients. The average Karnofsky score was 49, indicating poor performance status. The study found no association between the presence or absence of DVT and TPX use [6], questioning the role of TPX in this population. There was no difference in survival between those with or without DVT.

The problem, however, should not be underestimated, particularly, nowadays when the perspective on hospice care has been changing. In 2010, almost one-fifth of hospice patients were discharged home in the United States [12], whereas discharge rate to home from PCUs was shown to be as high as 39% [13], pointing to the increasing role of these institutions is not only providing terminal care but also in improving patients’ condition. This should be considered in the decision-making processes in these settings.

This paper reviewed the data on TPX in palliative care and hospice patients and summarized the updated recommendations on the TPX in this population.

## 2. Prevalence of Thromboprophylaxis in Hospices and Palliative Care Units

Thromboprophylaxis can be either primary—aimed at reducing the risk of VTE occurrence, or secondary—when the goal is to minimize the chances of recurrent VTE in patients with a known history of thrombosis. The prevalence of anticoagulation therapy at the end of life setting, both primary and secondary, varies across the institutions and countries. A study by Holmes et al. [14] showed that 9% (1557 out of 16,896) of lung cancer patients who were receiving home hospice care were prescribed TPX, although the type of TPX (primary vs. secondary) was not specified. Similar data were reported by Johnson et al. [15] in a retrospective study, demonstrating that primary TPX was being received by 6% of patients admitted to hospice in the UK. The use of TPX in hospice patients in another UK study was even lower, at 3.7% [16]. A retrospective cohort study by Kowalewska et al. [17] revealed that 6.7% of all (77 out of 1141) patients and 4.6% of cancer patients discharged from hospital to hospice care were prescribed antithrombotic therapy. It was shown that cancer patients, which constituted 60% of the study group, were significantly less likely to receive a prescription for anticoagulation, and the rationale for the de-escalation of TPX was increased bleeding risk, inconsistency with goals of care, or patient or family preference. 

With regards to PCU, a retrospective analysis at Genevan PCU showed that TPX was used in 43% of cancer patients [18]. Likewise, the TPX prevalence was high (44%) in a French study, which enrolled 1199 PCU patients, 91% of whom were cancer patients [19]. A cross-sectional study on the prevalence of TPX among 134 PCU patients in Austria revealed that primary and secondary TPX was used in 49% of cancer patients, similarly to non-cancer patients (42%) [20], although there was a tendency to discontinuation of TPX upon admission to PCU. A similar trend was also reported by Legault et al. [21] in a retrospective analysis, revealing TPX prevalence of 44% on admission to PCU, followed by 87.7% TPX discontinuation rate within 72 hours of admission. 

Although the data are limited, these results suggest that PCU patients are more likely to receive TPX compared to patients receiving hospice care (Table 1). It should be noted, however, that in some countries, e.g., United Kingdom, the terms “hospice” and “PCU” are synonymous; therefore, in those countries, active anti-cancer treatment and hospice care are not mutually exclusive. In contrast, in the United States, hospice patients usually no longer receive cancer-targeted therapies, and the vast majority hospice care is provided at home with the remainder of patients receiving hospice care in nursing homes or in-patient hospices, which are separated from acute care hospitals. These differences should be taken into account when comparing the data between the countries.

The relatively low prevalence of TPX and the trend to its discontinuation after transitioning to hospice might also result from palliative care providers’ belief that TPX should not be considered a priority in this setting, as shown in a qualitative study by Noble et al. [22]. The same study showed that, should TPX be proven effective in terms of symptom control, the providers were amenable to change of practice. A survey study among senior doctors in hospice inpatient units showed that although in 2000, 62% of physicians would stop TPX in patients with a high thrombotic risk who were intended for discharge home, in 2005, it was only 18% of respondents [23], suggesting an evolution of the approach to the TPX in this population.

## 3. Who Needs TPX?

Aside from its ethical aspect, clinical decision-making regarding TPX for hospice and palliative care cancer patients is challenging, also due to the heterogeneity of this population. Frequently, the patients may have contraindications to TPX, e.g., bleeding or thrombocytopenia [16], or the risks and benefits profile may be vague. A study by White et al. [6] revealed that previous VTE, being bedbound in the past 12 weeks, and lower limb edema were independent risk factors for VTE in PCU and hospice patients. 

The clinical status of the patient also plays an important role in making decisions on TPX. A survey study among experts in palliative care, oncology, intensive care, and anticoagulation on whether they would use TPX on a virtual palliative care patient showed that all physicians opted to withdraw TPX in patients with Karnofsky index less than 10 [24]. Tools designed to select hospice or palliative care patients, who would benefit from TPX the most, would significantly aid in the decision-making process. 

So far, the only available palliative-modified risk assessment tool is the pan Birmingham cancer network (PBCN) palliative-modified thromboembolic risk factors (THRIFT) score [15]. It includes a number of clinical risk factors to stratify patients to a high, intermediate, or low risk of VTE. The tool is not specific to cancer patients and was designed for use in a broadly defined palliative care population. A retrospective analysis of 1164 hospice inpatients in the U.K. revealed that a high/moderate THRIFT score had a high sensitivity (98.4%) but very low specificity (5.8%) in VTE risk prediction, suggesting the need of continued research in that matter.

Several models to predict the risk of VTE in cancer patients have been developed. Khorana score aims to identify ambulatory cancer patients at increased risk of VTE during chemotherapy [25]. It is a user-friendly tool based on routinely available predictive variables. Since this model has been tested in cancer patients receiving chemotherapy, it is questionable whether it should be applied to hospice or palliative care patients, the majority of whom do not continue active anti-neoplastic treatment. Moreover, 91.6% of patients in the study establishing Khorana score have shown eastern cooperative oncology group (ECOG) performance status of 0 to 1, whereas, in patients approaching the end of life, it is usually higher. Since the external validation of Khorana score has revealed a high proportion of patients falling into the intermediate-risk category (>50%) [26], several modifications of the Khorana score have been suggested, e.g., by addition of D-dimer and soluble P-selectin (Vienna score) [27]. However, a test for P-selectin is usually not available in routine clinical practice, making the use of this test infeasible. Another scoring system based on factors, such as Khorana score >2, previous VTE, metastatic disease, and vascular or lymphatic macroscopic compression, has been investigated in the ONKOTEV study [28] and has shown to have higher predictive power compared to Khorana score alone. Again, since the vast majority of patients in this trial were undergoing active anti-cancer treatment, the usefulness of the ONCOTEV score in PCU or hospice population not receiving cancer-targeted treatments is uncertain. 

Recently, another prediction model for cancer-associated VTE, incorporating only one clinical factor (tumor-site category) and one biomarker (D-dimer), has been proposed [29]. Due to its simplicity, it may have the potential to become a useful tool for the screening of hospice/palliative care patients at increased risk for VTE, which could aid with decision-making regarding TPX in this setting. So far, this score has been validated only on a cohort of cancer patients of whom the majority were undergoing chemotherapy; therefore, further studies are required to investigate its utility in terminally ill cancer patients who can no longer benefit from active treatment.

Ferroni et al. [30] introduced an interesting VTE risk assessment model, which uses a combination of machine learning and artificial intelligence to design a set of VTE predictors, exploiting certain patterns in demographic, clinical, and biochemical data for VTE risk stratification. This method, similarly to the above, has been validated only in a cohort of cancer patients undergoing chemotherapy; however, due to its low cost, non-invasiveness, and user-friendly approach, it may be also a promising tool for VTE risk assessment in hospice or palliative care patients not receiving active anticancer treatment.

## 4. Thromboprophylaxis Agent Selection

There are various TPX methods used in clinical practice. However, due to the complexity and uniqueness of palliative care and hospice patient population, TPX agent selection may be challenging.

Vitamin K antagonists (VKAs), e.g., warfarin and acenocoumarin, have been used for decades in the management of cancer-related VTE. Nowadays, however, their use in clinical practice has become limited due to multiple interactions with food and medications used in cancer treatment. Patients receiving VKA require frequent monitoring of the clotting time (international normalized ratio, INR), which are not only cumbersome for patients but may also decrease treatment compliance [31]. Additionally, INR has been shown to be more labile in patients under hospice or palliative care due to, e.g., a high prevalence of liver dysfunction and malnutrition; therefore, more frequent INR monitoring may be necessary for this population [32].

Low-molecular-weight heparin (LMWH) is recommended as the first-line treatment of VTE in cancer patients due to its superiority over warfarin in the prevention of recurrent VTE without an increase in major bleeding complications [33]. LMWH has fewer interactions with other drugs and generally does not require frequent blood monitoring. However, hospice patients often have low body weight and impaired renal function, in which cases blood monitoring may be necessary. The controversy around LMWH use in terminally ill patients arises due to the need for daily painful injections, which are not in line with the philosophy of palliative medicine. However, as reported by Noble et al. [34], LMWH was found to be an acceptable intervention by palliative care cancer patients, and the only negative experience was bruising. LMWH was shown to have little or no influence on the QoL, in contrast to anti-embolic stockings, which were found to negatively impact the QoL [34]. Additionally, the results of a recent qualitative study on the treatment of cancer-associated thrombosis demonstrated that although the patients found taking tablets easier, they preferred injected anticoagulants if found to be more effective than tablets [35].

Fondaparinux, an indirect inhibitor of factor Xa, is frequently recommended for patients having contraindications to LMWH [36]. However, due to its dependence on renal clearance, its use in patients with advanced malignancy may be limited. Similar to LMWH, it is administered by deep subcutaneous injections, which may be found bothersome by some patients.

Novel oral anticoagulants (NOACs), which are direct inhibitors of coagulation factor IIa (dabigatran) and Xa (e.g., rivaroxaban, edoxaban, apixaban), have gained significant attention in the last decade. Several trials have shown that NOACs are effective and safe for the treatment of VTE [37], and cancer patients-subgroup analysis of these trials has revealed that NOACs are non-inferior to VKA in cancer patients [38,39,40]. However, these studies have excluded patients with renal or hepatic function impairment, both of which are frequent conditions in palliative or hospice patients. The analysis of NOACs use for the treatment of VTE in patients with advanced cancer has found a 5.5% and 20% risk of major and non-major bleeding, respectively [41]. Poor performance status is an independent factor for increased risk of bleeding. Therefore, the use of NOACs for VTE treatment in patients in advanced stages of malignancy remains questionable. There may be, however, a role for these medications in the primary TPX. A meta-analysis of randomized controlled trials investigating the use of NOACs in a total of 13,338 cancer patients for primary TPX has revealed that NOACs are effective in VTE prevention and does not increase the risk of major bleeding compared to placebo [42], although a subgroup analysis of advanced cancer patients has not been performed. Although non-invasiveness and no need for monitoring would make NOACs convenient for use in palliative care and hospice, the actual use may be limited in this setting due to decreased oral intake. 

## 5. Current Recommendations 

Based on the most recent guidelines issued by National Institute for Health and Care Excellence (NICE) [36], VTE prophylaxis should be considered for patients receiving palliative care; however, factors, including temporary increases in thrombotic risk factors, risk of bleeding, estimated life expectancy, and the views of the patient and their family members or carers, should be taken into account. This is different from previous NICE guidelines in which TPX in palliative care is recommended only for patients who have potentially reversible acute pathology [43]. It is emphasized not to offer VTE prophylaxis to people in the last days of life. Additionally, VTE prophylaxis should be reviewed daily. NICE recommends LMWH as a first-line agent and fondaparinux in case of contraindications to LMWH [36].

The most updated 10th edition of antithrombotic guidelines issued by the American College of Chest Physicians (CHEST) does not refer to VTE prevention among palliative care patients [32], although in 8th edition, TPX is considered acceptable for carefully selected group of palliative care patients, i.e., in whom it could prevent worsening of the QoL [44].

Current National Comprehensive Cancer Network (NCCN) guidelines support lifelong secondary TPX for patients with active cancer and a history of VTE [9]. Although TPX in a palliative care setting is not directly referred to in the recommendations, factors to consider before implementing VTE prophylaxis include lack of palliative benefits or any unreasonable burden of TPX (e.g., painful injections or frequent monitoring with phlebotomy).

In the most recent guideline update, the American Society of Clinical Oncology (ASCO) does not comment on TPX in palliative care [45]. Of note, therapeutic anticoagulation (i.e., VTE treatment) is not recommended for patients for whom anticoagulation is of uncertain benefit, including patients receiving end-of-life/hospice care or those with very limited life expectancy with no palliative or symptom reduction benefit. Whether this approach can be extrapolated to TPX remains uncertain.

The European Society for Medical Oncology (ESMO) guidelines on VTE management and prophylaxis do not refer to hospice patients [46]. For cancer patients with a history of VTE who are treated with palliative chemotherapy in the metastatic setting, an indefinite secondary TPX should be discussed with patients.

There is also no reference to the palliative care population in 2019 international clinical practice guidelines for the treatment and prophylaxis of venous thromboembolism in patients with cancer [47]. Of note, TPX is recommended for hospitalized cancer patients with reduced mobility and should not be routinely used in ambulatory cancer patients, including those receiving systemic anticancer therapy.

The above recommendations are summarized in Table 2. To our knowledge, the NICE guidelines are the only ones specifically addressing TPX in hospices or palliative care units.

Due to the lack of large, randomized studies on TPX in this setting, providers have to rely on their own assessment and experience. It has also become a more common practice to implement internal institutional policies on TPX [23]. Terminally ill patients wish to and should be, whenever possible, involved in the decision-making process regarding TPX, particularly where the evidence-based guidelines are lacking [34].

## 6. Risks and Challenges

When considering TPX for cancer patients, increased risk of bleeding in this population remains an important issue. In a large, prospective study enrolling almost 3000 cancer patients, the abnormal renal function, metastatic disease, recent major bleeding, and recent immobility for more than 4 days were shown to be associated with a higher risk for both fatal PE and fatal bleeding [48]. Additionally, bodyweight <60 kg was an independent factor for fatal bleeding. Considering that hospice and palliative care patients frequently have a combination of these factors, bleeding risk in this subpopulation may be even higher, which may influence providers’ decisions on TPX. 

In the only one randomized study investigating prophylactic LMWH vs. placebo in 20 PCU patients with a life expectancy of ≤6 months, one VTE and one major bleeding occurred in the group receiving nadroparin (*p* = 1), whereas two minor bleedings occurred in the control group (*p* = 0.474) [11]. More light on bleeding risk in terminally ill PCU patients has been shed by a multicenter observational RHESO study [19]. Among twelve hundred patients on the study group, the majority of whom were cancer patients (91%), 44% were receiving primary or secondary TPX using LMWH or fondaparinux. The rate of clinically relevant bleeding, defined as a composite of a major bleeding and clinically relevant non-major bleeding, was 9.8% at 3 months. Bleeding occurred in 11% of patients who received TPX, and in 8.4% of those who did not, whereas the incidence of fatal bleeding was 2.1% vs. 1.8%, respectively. Cancer, recent bleeding, antiplatelet treatment, and TPX were found to be independent risk factors for clinically relevant bleeding, increasing the risk of the event 5.7, 3.4, 1.7, and 1.5 times, respectively. 

Discussions on the TPX in terminal care should also include cost analysis. It has been calculated that if all immobile cancer patients were to receive prophylactic LMWH, the expenses for medications of a hospice would increase by almost 30% [49]. Costs involved in the TPX and management of potential bleeding events may be difficult to overcome since a lot of hospices are reimbursed on a fixed per diem basis—particularly in the United States—or they are supported primarily by charities.

## 7. Conclusions

Introducing uniform guidelines on TPX at the end of life care is encouraged. Ideally, they should be based on the results of clinical trials, focusing on this group of patients. The patient population should be carefully described with regard to the stage of the disease, goals of treatment, and nearness to the very end of life. The development of tools to predict VTE in this patient population would aid with decision-making regarding TPX. Although the risk of anticoagulation cannot be underestimated, there may be a group of patients who would benefit from symptomatic relief of TPX. Finally, the results of White et al. study [6] significantly challenge the appropriateness of TPX in advanced cancer patients with poor performance status, who are nearing the end of life.

## Figures and Tables

**Table 1:** Thromboprophylaxis (TPX) prevalence in hospice and palliative care units (PCU).

Authors	Thromboprophylaxis (TPX) Prevalence % (Number of Patients Receiving TPX/all Patients)	Type of TPX (Primary/Secondary)	% of Cancer Patients in the Study Group	Setting
Holmes et al. [14]	9 (1557/16,896)	No data	100	Hospice
Johnson et al. [15]	6 (68/1164)	Primary	82	Hospice
Gillon et al. [16]	3.7 (13/350)	Primary	77	Hospice
Kowalewska et al. [17]	4.6 (31/674)	Primary and secondary	100	Hospice
Pautex et al. [18]	43 (103/240)	No data	100	PCU
Tardy et al. [19]	44 (527/1199)	Primary	91	PCU
Gartner et al. [20]	49 (56/115)	Primary and secondary	100	PCU
Legault et al. [21]	44 (56/127)	Primary	92	PCU

**Table 2:** Summary of guidelines for thromboprophylaxis in the palliative care setting.

Recommendation	Author	References
Thromboprophylaxis (TPX) should be considered for patients receiving palliative care; however, factors, including temporary increases in thrombotic risk factors, bleeding risk, estimated life expectancy, and the views of the patient and their family/carers, should be taken into account. TPX should not be offered to patients in the last days of life. TPX should be reviewed daily.	National Institute for Health and Clinical Excellence (NICE)	[36]
No guidelines on TPX in palliative care.	American College of Chest Physicians (ACCP)	[32]
No guidelines on TPX in palliative care. Before implementing VTE prophylaxis in all patients, factors to consider include lack of palliative benefits or any unreasonable burden of TPX.	National Comprehensive Cancer Network (NCCN)	[9]
No guidelines on TPX in palliative care.	American Society of Clinical Oncology (ASCO)	[45]
No guidelines on TPX in the hospice setting. Secondary TPX should be discussed with patients receiving palliative chemotherapy.	European Society for Medical Oncology (ESMO)	[46]
No guidelines on TPX in palliative care.	International clinical practice guidelines	[47]
